# Effects of different injection methods of propofol anesthesia on the behavior and electroencephalography recording in mice

**DOI:** 10.1002/ibra.12030

**Published:** 2022-03-08

**Authors:** Dan Luo, Shi‐Yu Chen, Yu Zhang

**Affiliations:** ^1^ College of Anesthesiology Zunyi Medical University Zunyi Guizhou China; ^2^ Guizhou Key Laboratory of Anesthesia and Organ Protection Zunyi Medical University Zunyi Guizhou China

**Keywords:** electroencephalography, general anesthesia, loss of consciousness, propofol, recovery of consciousness

## Abstract

Propofol is commonly used in mice studies on the mechanism of general anesthesia. The administration routes of propofol include intraperitoneal injection, single tail vein injection, and continuous tail vein pumping. The aim of this study is to compare the effects of the three injection methods on the behavior and electroencephalography (EEG) recording in mice. Mice were divided into an intraperitoneal injection group, a single tail vein injection group, and a continuous tail vein pumping group according to the propofol administration route. The indexes for observation were: time of loss of righting reflex (LORR), time of resumption of righting reflex (RORR), and change in the number of EEG spindle waves during anesthesia. The LORR and RORR were detected again after 1 week to determine the repeatability of the three administration routes. Death and behavioral change after anesthesia recovery in mice were recorded in the three groups. For propofol administration in mice, intraperitoneal injection induced long‐duration anesthesia, but the depth of anesthesia was shallow and there was a risk of anesthesia accidents. A small dose of propofol administered through a single tail vein can induce loss of consciousness but the LORR time was not recorded, hence the metrics during induction of anesthesia were not investigated. Continuous tail vein pumping produced stable behavior and EEG recording during anesthesia induction and recovery in mice, and the individual difference was small. Continuous tail vein pumping is an ideal administration route for studying the mechanism of loss of consciousness of propofol anesthesia in mice, which could provide reference data for future mice experiments using propofol.

## Graphical abstract

- Intraperitoneal injection of propofol can collect the time and electroencephalography (EEG) of loss of righting reflex (LORR) and RORR, but it has operational interference, high mortality, and poor repeatability.
- Single tail vein injection of propofol has high repeatability and low mortality, but only the time and EEG of RORR can be collected, and there is operational interference in the experiment.
- Continuous tail vein pumping of propofol can collect the time and EEG of LORR and RORR, with no operational interference, low mortality, and high repeatability in the experiment.

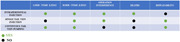

## 1 INTRODUCTION

Anesthesia is a necessary prerequisite for surgery. Early anesthesia methods almost completely rely on gas inhalation anesthetics, such as ether, chloroform, and nitrous oxide, which have slow anesthetic effects, slow recovery of consciousness, and postoperative adverse reactions. With the development of technology, intravenous anesthesia is gradually applied in clinical practice, and it is characterized by quicker effects and fewer postoperative adverse reactions compared with inhalation anesthesia. One of the most representative of intravenous anesthesia is thiopental sodium. Thiopental sodium, as a potent barbiturate, can rapidly induce anesthesia, but due to its slow metabolism, multiple dosing will significantly increase the risk of delayed recovery of consciousness in patients.^1^ To solve this problem, propofol can come in handy; it is an intravenous hypnotic drug used to induce and maintain sedation and general anesthesia, which works by enhancing the inhibitory neurotransmitter γ‐aminobutyric acid (GABA) on the GABAA receptor.^2^ Its properties include rapid metabolism, multiple doses that do not markedly increase the risk of delayed recovery of consciousness, and a low incidence of postoperative nausea, which makes it widely used in clinical practice.^3^ In 2018, Dr. John Glenn, a pioneer in the discovery and clinical development of propofol, was awarded the Lasker Clinical Medicine Research Award in the United States, which suggests that propofol exerts a crucial role in clinical anesthesia.^4^ The research on the mechanism of general anesthesia is always inescapable on account of the importance of propofol.

Due to the limitations of clinical studies, experimental animals, especially mice, have become vital research objects in the study of the mechanism of general anesthesia.^5^ The time of loss righting reflex (LORR) can represent the anesthesia induction time, and the time of resumption of righting reflex (RORR) can represent the anesthesia recovery time.^6^ The anesthesia induction and recovery time only reflect the behavioral changes of mice, while the electroencephalography (EEG) in anesthesia induction and recovery can objectively respond to the change of anesthesia depth.^7^ The key to the success of the experiment is to avoid the death of experimental animals and ensure the reproducibility of positive results.

In view of the fact that propofol is the most widely used general anesthetics in clinics and the representative of intravenous general anesthetics combined with the mice to study the mechanism of general anesthesia. In addition, the propofol administration route may influence the anesthesia process in mice, and there is still no comprehensive or systematic study for this. Therefore, to clarify the effect of three administration routes commonly used at present (intraperitoneal injection,^8^ single tail vein injection,^9^ and continuous tail vein pumping^10^) on the anesthesia effect, mice were used as experimental animals and propofol was used as an anesthesia drug to study the behavior and EEG changes under propofol anesthesia. Meanwhile, this study compared and evaluated the three propofol administration routes for feasibility and repeatability with LORR time, RORR time, and the number of EEG spindles as indicators, so as to provide a reference for future experiments of mice using propofol.

## 2 MATERIALS AND METHODS

### 2.1 Laboratory animals

Healthy male adult C57BL/6J mice, aged 8–12 weeks (body weight 20–25 g) with specific pathogen‐free (SPF) grade, were purchased from Hunan SJA Laboratory Animal Co., Ltd. (Certificate No.: SCXK [Hunan] 2019‐0004). The animals were fed in the SPF Laboratory Animal Room of Guizhou Provincial Key Laboratory of Basic Research of Anesthesia and Organ Protection & Brain Science. They had free access to water and food, and were allowed to move freely, and for the SPF Laboratory Animal Room, a 12 h (19:00–07:00) light/12 h (07:00–19:00) dark cycle was adopted, with a room temperature of 25 ± 2°C, relative humidity 55 ± 5%, and noise <50 dB. The feeding and use of animals were approved by the Animal Management Committee of the Ethics Committee of Zunyi Medical University in accordance with the National Law on the Use of Experimental Animals.

### 2.2 Reagents and instruments

Reagents and instruments used in this study were as follows: propofol injection (20 ml: 0.2 g; Xi'an Libang Pharmaceutical Co., Ltd., Batch No.: 21911202), isoflurane (RWD Life Science Co., Ltd.), stereotaxic instrument for mice (RWD Life Science Co., Ltd.), rocker microscope (QAMHG‐2400L; Thinker Tech Nanjing Biotech Co., Ltd.), anesthesia induction chamber for mice (RWD Life Science Co., Ltd.), isoflurane anesthesia gas vaporizer (RWD Life Science Co., Ltd.), electroencephalography system (Bio‐Signal), mouse tail vein injector (Zhongke Life Science), microinjection pump (WZS‐50F6; RWD Life Science Co., Ltd.), and electronic balance (BS224S; Sartorius GMBH).

### 2.3 Implantation of cortical EEG electrode

First, the mice were anesthetized by isoflurane and fixed on the stereotaxic instrument. Then their scalps were cut to open and fully expose the skulls, and the bregma point and lambda point were adjusted to the same level. After that, four cranial peg screws were fixed to the skull surface, respectively, according to coordinates (anterior–posterior [AP]: +1, medial–lateral [ML]: ±1.5; AP: −3.5, ML: ±1) and then the EEG electrode was installed. The connections between the cranial peg screws and the electrode were adhered by the 1454 instantaneous, and the skull surface was covered with a self‐adhesive dental cement to fix the electrode. The mice were placed in an incubator until their revival after the coagulation of dental cement. Subsequently, the propofol injection experiment was performed 1 week after the mice were recovered.

### 2.4 Grouping and administration

Thirty mice implanted with EEG electrodes successfully were randomized into three groups: intraperitoneal injection group, single tail vein injection group, and continuous tail vein pumping group, with 10 mice for each group. For the intraperitoneal injection group, the EEG of awake mice was recorded for 5 min, and then the mice were pinched on the back of the skin with the left hand, with the abdomen up and the head tilted down at a 45° angle. With the right hand holding the syringe, the propofol was injected into the abdominal cavity of the mice within 2 s with a dose of 100 mg/kg, and then placed in a cage for observation. For the single tail vein injection group, the EEG of awake mice were recorded for 5 min, and then with the aid of tail vein fixator, the propofol was injected into the tail vein within 1 s with a dose of 20 mg/kg, and then mice were placed in a cage for observation. For the continuous tail vein pumping group, the EEG adapter was connected with the EEG on the top of the mouse's head and placed in the tail vein fixator. A syringe needle for propofol injection was inserted into the tail vein and fixed it properly, and then connected with the microinjection pump. The EEG data were recorded 5 min before administration, and the propofol was pumped continuously at a rate of 10 mg/kg/min until LORR. The relevant data were observed.

The following were the relevant recording indexes: ① LORR time: For the intraperitoneal injection group and single tail vein injection group, it referred to the time from the completion of propofol administration to the point when the mice were lied flat, could not turn over and touch the ground on all fours, while for the continuous tail vein pumping group, it referred to the time from the beginning of propofol pumping to the point when the mice lie flat, cannot turn over and touch the ground on all fours. ② RORR time: It referred to the time from LORR to the point when the mice could turn over, and touch the ground on all fours. ③ Cortical EEG spindles: it referred to high amplitude brainwaves with a frequency of 10–15 Hz and a duration greater than 0.5 s, which occurred frequently during the loss of consciousness for propofol anesthesia, reflecting the propofol anesthesia time in mice objectively.^11^, ^12^ LORR and RORR time were recorded again for evaluating the repeatability of the three administration routes in the study on propofol anesthesia in mice 1 week after the first experiment in 30 mice. ④ Death and behavioral change after anesthesia recovery.

### 2.5 Statistical analysis

GraphPad Prism 8.0 and Spike 8.2 were used for statistical analysis and plotting. The experimental data were expressed as mean ± standard deviation (mean ± SD). One‐way analysis of variance was used for an intergroup comparison, while paired *t* test for intragroup comparison. The significant difference was presented as *p* < 0.05.

## 3 RESULTS

### 3.1 Effects of different propofol administration routes on LORR and RORR times in mice

The LORR and RORR times of mice under different propofol administration routes are shown in Table 1. For the intraperitoneal injection group, LORR time in the first and second experiments (1 week after the first experiment) were 386.90 ± 81.36 s (*n* = 10) and 259.33 ± 57.51 s (*n* = 9, a mouse died in the second experiment), respectively, indicating a significant difference between the two experiments (*p* < 0.05); RORR time in the first and the second experiments were 715.80 ± 214.92 s (*n* = 10) and 4208.33 ± 1377.34 s (*n* = 9), respectively, with a significant difference (*p* < 0.01). For the single tail vein injection group, LORR appeared immediately after injection of propofol due to the rapid effect of anesthesia, and so, the LORR time could not be recorded; the RORR time in the first and second experiments were 195.60 ± 17.96 s (*n* = 10) and 196.90 ± 29.57 s (*n* = 10), respectively, with no significant difference (*p* > 0.05). For the continuous tail vein pumping group, LORR time in the first and second experiments were 150.80 ± 12.27 s (*n* = 10) and 157.00 ± 16.67 s (*n* = 10), respectively, with no significant difference (*p* > 0.05); the RORR time in the first and second experiments were 156.50 ± 28.26 s (*n* = 10) and 150.40 ± 21.26 s (*n* = 10), respectively, with no significant difference (*p* > 0.05).

**Table 1:** LORR and RORR times of mice under different propofol administration routes

	First experiment		Second experiment	
Administration route	LORR time (s)	RORR time (s)	LORR time (s)	RORR time (s)
Intraperitoneal injection	386.90 ± 81.36	715.80 ± 214.92	259.33 ± 57.51*	4208.33 ± 1377.34**
Single tail vein injection	Immediately	195.60 ± 17.96	Immediately	196.90 ± 29.57
Continuous tail vein pumping	150.80 ± 12.27	156.50 ± 28.26	157.00 ± 16.67	150.40 ± 21.26

*Note*: For the comparison between the first and second experiments, *p < 0.05, **p < 0.01 (*n* = 10).

Abbreviations: LORR, loss righting reflex; RORR: resumption of righting reflex.

### 3.2 Effects of different propofol administration routes on cortical EEG in mice

**Figure 1:**
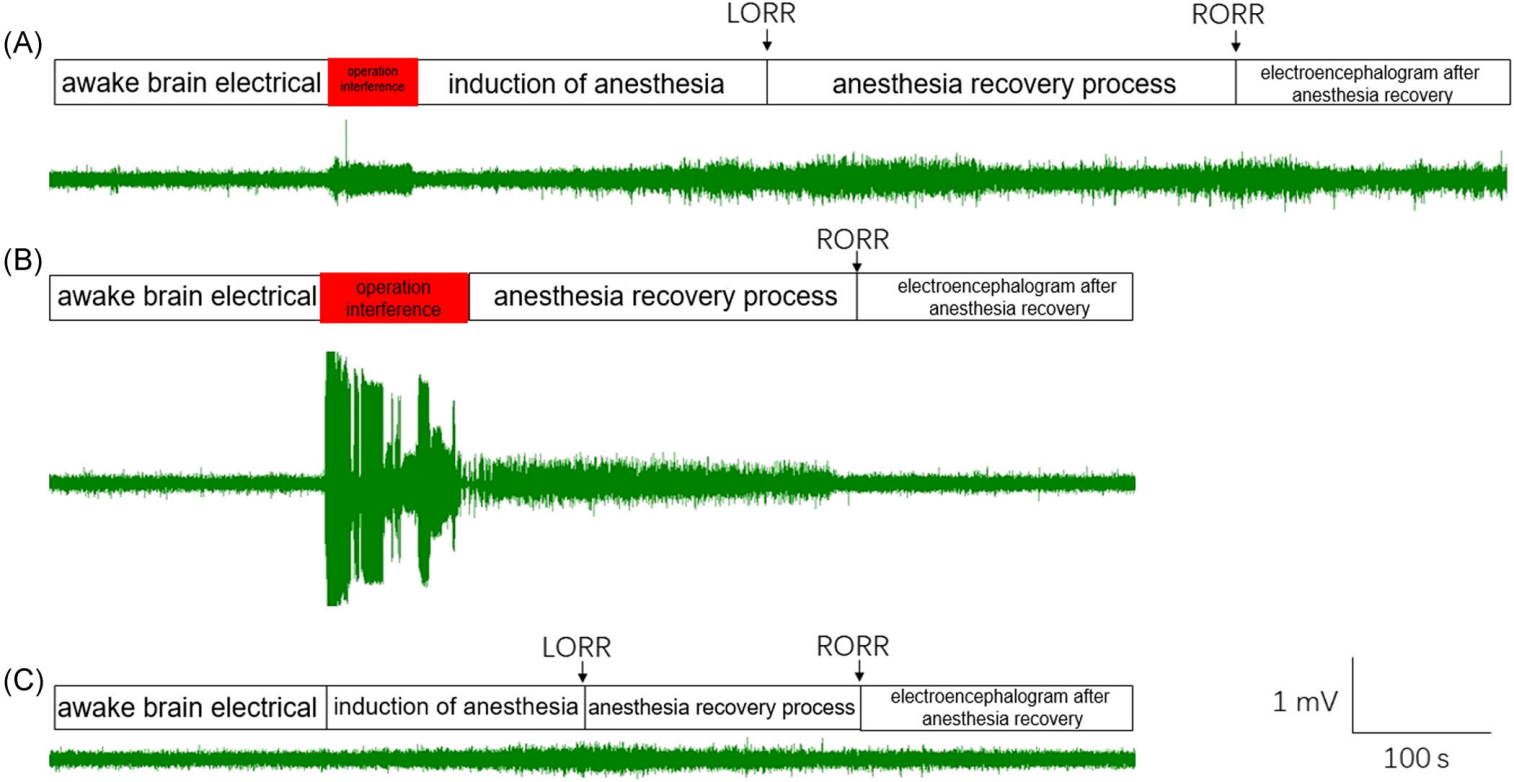
Comparison of original cortical EEG during propofol administration between the three groups of mice. (A) In the intraperitoneal injection group, the cortical EEG of mice from LORR to RORR could be completely recorded, the red area was operational interference. (B) In the single tail vein injection group, the red area was operational interference and the cortical EEG in the LORR period could not be recorded. (C) In the continuous tail vein pumping group, the cortical EEG of mice from LORR to RORR could be completely recorded. EEG, electroencephalography; LORR, loss righting reflex; RORR, resumption of righting reflex [Color figure can be viewed at wileyonlinelibrary.com]

In the intraperitoneal injection group, the cortical EEG of mice from LORR to RORR could be completely recorded for the intraperitoneal injection group, but grasping, pinching, and the injection operation interfered with the EEG significantly. Moreover, after RORR, the mice also repeatedly experienced low‐frequency and high‐amplitude EEG for low‐level consciousness, which was similar to that during anesthesia (Figure 1A). In the single tail vein injection group, LORR appeared immediately after single tail vein injection, and the cortical EEG in the LORR period could not be recorded, herein, only the cortical EEG in the RORR period was recorded. However, the single tail vein injection needed to be performed in a tail vein fixator, which restricted the activity of mice and caused significant interference to the EEG. In addition, after RORR, the EEG was restored to low‐amplitude and high‐frequency EEG for awake mice (Figure 1B). In the continuous tail vein pumping group, the cortical EEG during LORR and RORR periods was recorded completely without interference. After RORR, the EEG was restored to low‐amplitude and high‐frequency EEG for awake mice (Figure 1C).

### 3.3 Change in the number of cortical EEG spindles for different propofol administration routes

**Figure 2:**
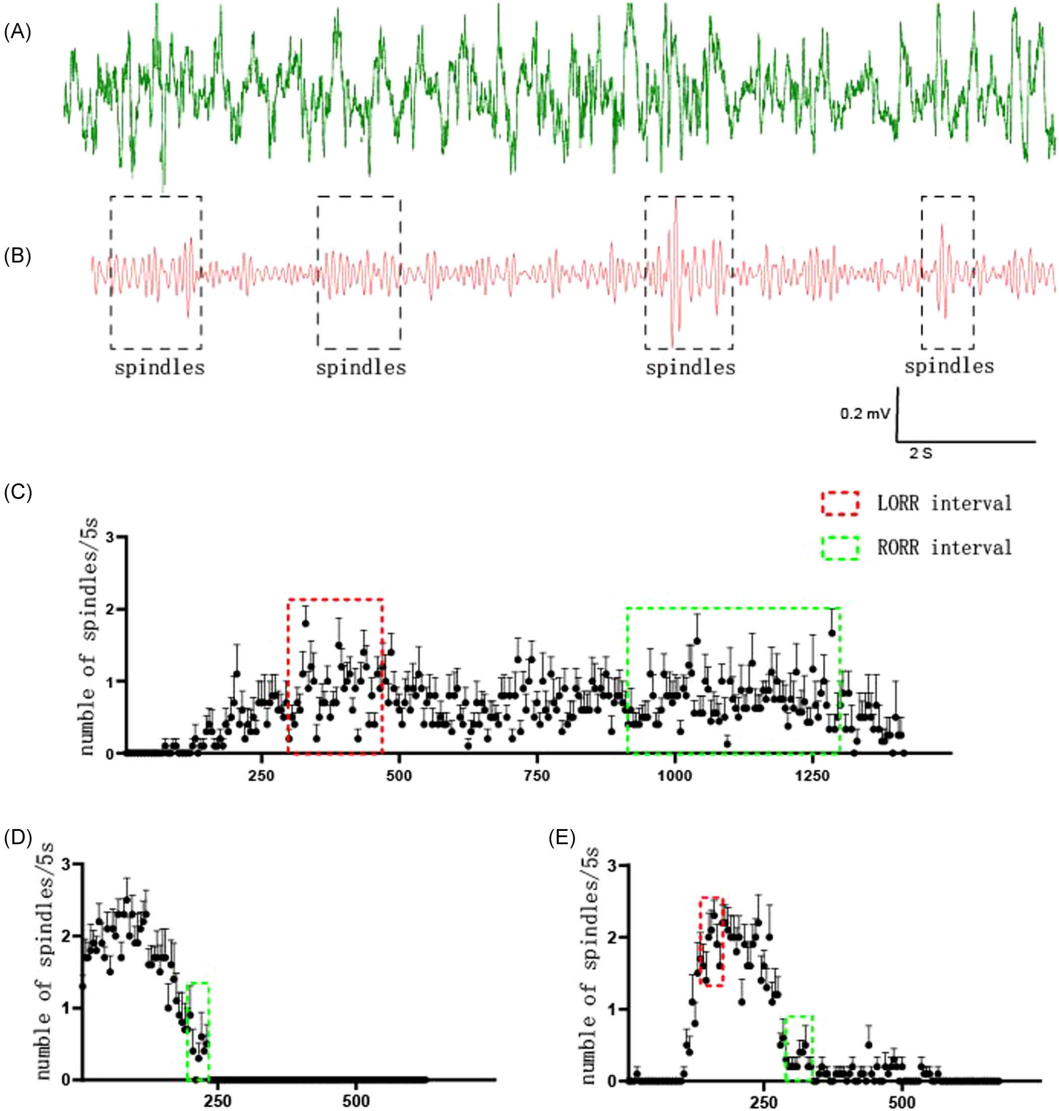
Change in the number of cortical EEG spindles for different propofol administration routes. (A) The original EEG in propofol anesthesia. (B) The original EEG in propofol anesthesia after filtering (10–15 Hz), the spindles presented in the black dotted line frame. (C) Change in the number of cortical EEG spindles for the intraperitoneal injection group (*n* = 10). (D) Change in the number of cortical EEG spindles for the single tail vein injection group (*n* = 10). (E) Change in the number of cortical EEG spindles for the continuous tail vein pumping group (*n* = 10). EEG, electroencephalography; LORR, loss righting reflex; RORR, resumption of righting reflex [Color figure can be viewed at wileyonlinelibrary.com]

In propofol anesthesia, the original EEG (green) was shown in Figure 1A. After 10–15 Hz of filtering, the original EEG (red) displayed typical spindles (black, in the dotted line frame, with the feature of thick in the middle and thin at both ends) (Figure 1B). Furthermore, in the intraperitoneal injection group, the spindles began to appear and increase slowly after 90 s of propofol administration. The number of spindles reached the upper limit (no more than 1.5/5 s) in the LORR interval (the shortest intragroup LORR time to the longest intragroup LORR time) and decreased slowly after the RORR interval (the shortest intragroup RORR time to the longest intragroup RORR time), but still lasted for more than 300 s. The total duration of the spindles exceeded 1500 s (Figure 2C). In the single tail vein injection group, the spindles appeared immediately after administration. The peak number of spindles, more than 2/5 s, reached within 90 s, then decreased rapidly until the RORR interval, where no spindle appeared again. Spindles lasted for no more than 250 s (Figure 2D). In the continuous tail vein pumping group, spindles appeared about 90 s after administration. The peak number of spindles reached in the LORR interval. The number of spindles was maintained at more than 2/5 s after LORR, and then gradually decreased until RORR. However, sporadic spindles still appeared after RORR, and the total duration of spindles did not exceed 600 s (Figure 2D).

### 3.4 Death and behavioral change after anesthesia recovery in mice for different propofol administration routes

There was no death in the single tail vein injection group or continuous tail vein pumping group (*n* = 10), but in the intraperitoneal injection group, there were two mice that died: One died during the recovery of consciousness in the second experiment (*n* = 10), and the other died 1 day after the second experiment (*n* = 10). Besides, after RORR, the mice in the intraperitoneal injection group presented with different degrees of drowsiness and tottered after stimulation (*n* = 10). In the single tail vein injection and continuous tail vein pumping groups, the mice showed no obvious drowsiness and did not totter after RORR (*n* = 10).

## 4 DISCUSSION

Propofol, as the main drug to study the mechanism of general anesthesia, has been widely used in experiments. However, different administration methods of propofol can cause different behavior and EEG changes in mice. To clarify the advantages and disadvantages of different administration methods, this experiment was designed to study the effects of intraperitoneal injection, single tail vein injection, and continuous tail vein pumping on mice. According to the preliminary experimental results, LORR appeared in all the mice, after intraperitoneal injection of propofol, only when the dose reached 100 mg/kg (20 mg/kg is required for a single injection of the caudal vein), which was consistent with relevant literature.^8^, ^9^ In the continuous tail vein pumping group, propofol was pumped at a speed of 10 mg/kg/min to meet the requirements for collecting data concerning LORR time and EEG, but the LORR of mice was only about 2.5 min, which was different from 5.5 min reported in a previous study.^10^ The difference in LORR time might be related to the temperature, circadian rhythm, and LORR criteria for the experiment,^13^ but this does not affect the experimental research.

According to the experimental results, the loss of consciousness after anesthesia was achieved by all of the three administration routes. Whereas in the intraperitoneal injection group without any interference, the LORR time in the second experiment was shorter than that in the first experiment, but the RORR time was significantly longer than that in the first experiment, suggesting that the first intraperitoneal injection of propofol might lead to celiac inflammatory hyperplasia and formation of new blood capillaries, which could accelerate the absorption of propofol and advance the anesthesia effect. Thus, the induction time was shortened and anesthesia recovery time was prolonged.^14^ Therefore, this administration route cannot be used for self‐controlled experiments for poor repeatability. Compared with the group of single tail vein injection and continuous tail vein pumping, the time of anesthesia maintenance was longer after intraperitoneal injection of propofol. The reason for this might be due to the higher dose of propofol for intraperitoneal injection. After injection into the abdominal cavity, propofol needed to be absorbed through the omentum majus into the blood circulation to work, but the absorption of omentum majus was slow.^15^ In this experiment, two mice in the intraperitoneal injection group died; the autopsy showed that one mouse died of glossocoma due to the prolonged recovery time of anesthesia, and the other died of intestinal paralysis caused by partial injection of propofol into the intestinal canal. This is because intraperitoneal injection of propofol may prolong the anesthesia time. The longer the anesthesia time, the higher the risk of hypothermia, hypoxia, and asphyxia in mice, which, in turn, increased their mortality.

In the single tail vein injection group, the LORR time was extremely short because a large amount of propofol entered the blood circulation through the tail vein rapidly to produce anesthesia effect. LORR appeared immediately after propofol injection for all of the mice; therefore, the LORR time and EEG data in the period for the mice could not be recorded, which is the greatest disadvantage of single tail vein injection. However, the dose for LORR induction was the lowest in the single tail vein injection group, and there was no spindle appeared after RORR, which is a typical manifestation of rapid metabolism of propofol anesthesia. Through this administration route, the recovery time of anesthesia is short, the risk of anesthesia complications is low, and the effect on physiological function of mice is small. Therefore, for a general anesthesia mechanism experiment without the requirement for LORR data, the single tail vein injection is a better administration route for propofol.

In different studies on the mechanism of general anesthesia, LORR and RORR times may be affected by subjective factors, leading to great differences in results, while the cortical EEG data during general anesthesia may reflect the change in consciousness level objectively.^16^ Therefore, EEG analysis is an important part of studies on the mechanism of general anesthesia. The EEG results have shown that the behavioral and EEG change during the induction and recovery of propofol anesthesia may be recorded continuously and completely without interference in the continuous tail vein pumping group, and the depth of anesthesia (with the upper limit for the number of spindles as the indicator) may reach the level in the single tail vein injection group, thus continuous tail vein pumping is an ideal administration route of propofol in studies on the mechanism of general anesthesia. Nevertheless, in the continuous tail vein pumping group, sporadic spindles still occurred after RORR in mice, indicating that the influence of continuous tail vein pumping on the mental functions of mice was greater than that of single tail vein injection, which may be associated with the larger dose and longer administration time of propofol.

The EEG analysis has shown that spindles are a characteristic brain waveform during nonrapid eye movement (REM) sleep, also known as sleep waves.^17^ The EEG during propofol anesthesia is similar to that during non‐REM sleep. Increasing evidence has shown that the deeper the depth of propofol anesthesia, the more the number of spindles. Therefore, during propofol anesthesia, the change in the number of spindles often reflects the depth of anesthesia.^12^ An analysis on the cortical EEG spindles found that in the intraperitoneal injection group, spindles began to appear about 90 s after administration, and the duration of spindles was more than 1500 s, but the number of spindles did not exceed 1.5/5 s, indicating that the duration of anesthesia was long but the depth of anesthesia was shallow. In the single tail vein injection group, spindles appeared immediately after administration. However, As 20 mg/kg propofol injection by single tail vein can cause explosive inhibition of EEG in some mice in the initial 90 s, the number of spindles gradually increased until more than 2/5 s, and then the number gradually decreased with the metabolism of propofol until no spindle appeared after RORR. The whole process lasted for no more than 250 s. In the continuous tail vein pumping group, no spindle appeared until about 90 s after administration. In the LORR interval, the number of spindles gradually increased until more than 2/5 s, and the number was maintained for about 90 s, and then gradually decreased until the appearance of RORR. After RORR, there were still sporadic spindles occasionally, and the whole process lasted for no more than 600 s. The reason for this EEG feature might be that the duration of continuous tail vein pumping was longer than that of a single injection, which resulted in a small amount of propofol remaining in the body after RORR.

The biggest advantage of propofol intraperitoneal injection is a simple operation, so this method can be widely used in the experiment of anesthesia depth and anesthesia process without strict requirements. For example, it is widely used to study the effects of propofol on sleep and awakening,^18^ memory,^19^ and other physiological functions. The only disadvantage of propofol single tail vein injection is the lack of data on LORR processes. Therefore, when only RORR processes need to be collected, the single tail vein injection of propofol is the optimal choice. Continuous tail vein pumping produced stable behavior and EEG recording during anesthesia induction and recovery in mice. In addition, the repeatability of the experiment is good and the animal mortality is low. As a result, continuous tail vein pumping is an ideal administration route for studying the mechanism of loss of consciousness of propofol anesthesia in mice.

## 5 CONCLUSION

In summary, in the intraperitoneal injection group, anesthesia time was long and shallow, the incidence rate of anesthesia accidents was high, repeatability was poor, and the required sample size was large, but the operation for administration is relatively simple. In the single tail vein injection group, the LORR time was too short to record relevant data during anesthesia induction, but the repeatability was good, and the dose for administration was small. The continuous tail vein pumping may well make up for the disadvantages of the above two administration routes, hence it is more conducive to study the general anesthesia mechanism of propofol. However, there are also some shortcomings for the continuous tail vein pumping: Relevant operation is more complicated than that of intraperitoneal injection and single tail vein injection, and the needle tends to easily fall off during initial operation, thus repeated practice is needed.

## CONFLICTS OF INTEREST

The authors declare no conflicts of interest.

## ETHICS STATEMENT

All animal operations were approved by the Animal Experiment Ethics Committee of Zunyi Medical University (Approval No. KLLY(A)‐2020‐034). All experiments conformed to the Guide for the Care and Use of Laboratory Animals published by the US National Institutes of Health.

## AUTHOR CONTRIBUTIONS

Yu Zhang and Dan Luo contributed to the central idea. Dan Luo and Shi‐Yu Chen conceived and designed the experiments. Dan Luo analyzed most of the data. Dan Luo wrote the initial draft of the paper. Yu Zhang and Dan Luo contributed to refining the ideas, carrying out additional analyses, and finalizing the paper.

## Data Availability

Data are available on request from the authors. The data that support the findings of this study are available from the corresponding author upon reasonable request.

## References

[1] Walsh CT . Propofol: milk of amnesia. Cell. 2018;175(1):10‐13. 10.1016/j.cell.2018.08.031 30217361

[2] Sahinovic MM , Struys M , Absalom AR . Clinical pharmacokinetics and pharmacodynamics of propofol. Clin Pharmacokinet. 2018;57(12):1539‐1558. 10.1007/s40262-018-0672-3 30019172 PMC6267518

[3] Glen JBI . The discovery and development of propofol anesthesia: the 2018 Lasker‐DeBakey Clinical Medical Research Award. JAMA. 2018;320(12):1235‐1236. 10.1001/jama.2018.12756 30208399

[4] Dankoski E . The 2018 Lasker~DeBakey Clinical Medical Research Award recognizes John Baird Glen for the discovery of propofol. J Clin Invest. 2018;128(10):4198‐4200. 10.1172/JCI124375 30199850 PMC6159986

[5] Hohlbaum K , Bert B , Dietze S , Palme R , Fink H , Thone‐Reineke C . Systematic assessment of well‐being in mice for procedures using general anesthesia. J Vis Exp. 2018;(133):57046. 10.3791/57046 29630060 PMC5933230

[6] Kitamura Y , Hongo S , Yamashita Y , et al. Influence of lipopolysaccharide on diazepam‐modified loss of righting reflex duration by pentobarbital treatment in mice. Eur J Pharmacol. 2019;842:231‐238. 10.1016/j.ejphar.2018.10.049 30391741

[7] Gui H , Liu C , He H , Zhang J , Chen H , Zhang Y . Dopaminergic projections from the ventral tegmental area to the nucleus accumbens modulate sevoflurane anesthesia in mice. Front Cell Neurosci. 2021;15:671473. 10.3389/fncel.2021.671473 33994950 PMC8119636

[8] Alves HC , Valentim AM , Olsson IA , Antunes LM . Intraperitoneal propofol and propofol fentanyl, sufentanil and remifentanil combinations for mouse anaesthesia. Lab Anim. 2007;41(3):329‐336. 10.1258/002367707781282767 17640460

[9] Cai S , Tang AC , Luo TY , et al. Effect of basal forebrain somatostatin and parvalbumin neurons in propofol and isoflurane anesthesia. CNS Neurosci Ther. 2021;27(7):792‐804. 10.1111/cns.13635 33764684 PMC8193699

[10] Wang YL . The Critical Role of Paraventricular Thalamic Nucleus on Regulating Loss of Consciousness Induced by Propofol. Wannan Medical College; 2020. 10.27374/d.cnki.gwnyy.2020.000013

[11] Fernandez LMJ , Luthi A . Sleep spindles: mechanisms and functions. Physiol Rev. 2020;100(2):805‐868. 10.1152/physrev.00042.2018 31804897

[12] Xi C , Sun S , Pan C , Ji F , Cui X , Li T . Different effects of propofol and dexmedetomidine sedation on electroencephalogram patterns: wakefulness, moderate sedation, deep sedation and recovery. PLoS ONE. 2018;13(6):e0199120. 10.1371/journal.pone.0199120 29920532 PMC6007908

[13] Shen JH , Ye M , Chen Q , et al. Effects of circadian rhythm on Narcotrend index and target‐controlled infusion concentration of propofol anesthesia. BMC Anesthesiol. 2021;21(1):215. 10.1186/s12871-021-01445-z 34488646 PMC8419887

[14] Wu J , Li C , Yuan W . Effects of Shenfu injection on macrocirculation and microcirculation during cardiopulmonary resuscitation. J Ethnopharmacol. 2016;180:97‐103. 10.1016/j.jep.2016.01.027 26806577

[15] Suarez‐Martinez AD , Peirce SM , Isakson BE , et al. Induction of microvascular network growth in the mouse mesentery. Microcirculation. 2018;25(8):e12502. 10.1111/micc.12502 30178505 PMC7446122

[16] Shin HW , Kim HJ , Jang YK , et al. Monitoring of anesthetic depth and EEG band power using phase lag entropy during propofol anesthesia. BMC Anesthesiol. 2020;20(1):49. 10.1186/s12871-020-00964-5 32102676 PMC7045415

[17] Kim A , Latchoumane C , Lee S , et al. Optogenetically induced sleep spindle rhythms alter sleep architectures in mice. Proc Natl Acad Sci USA. 2012;109(50):20673‐20678. 10.1073/pnas.1217897109 23169668 PMC3528529

[18] Yue XF , Wang AZ , Hou YP , Fan K . Effects of propofol on sleep architecture and sleep‐wake systems in rats. Behav Brain Res. 2021;411:113380. 10.1016/j.bbr.2021.113380 34033853

[19] Liang C , Du F , Wang J , Cang J , Xue Z . Propofol regulates neural stem cell proliferation and differentiation via calmodulin‐dependent protein kinase II/AMPK/ATF5 signaling axis. Anesth Analg. 2019;129(2):608‐617. 10.1213/ANE.0000000000003844 30303867

